# Determining the Optimal Sequence of Multiple Tests

**DOI:** 10.1002/sim.70254

**Published:** 2025-10-17

**Authors:** Lucas Böttcher, Stefan Felder

**Affiliations:** ^1^ Department of Computational Science and Philosophy Frankfurt School of Finance and Management Frankfurt am Main Germany; ^2^ Laboratory for Systems Medicine, Department of Medicine University of Florida Gainesville Florida USA; ^3^ Faculty of Business and Economics University of Basel Basel Switzerland; ^4^ Faculty of Business and Economics University of Duisburg‐Essen Essen Germany

**Keywords:** combination testing, diagnostic tests, optimal testing, receiver operating characteristics, test threshold, treatment threshold, value of information

## Abstract

The use of multiple tests can improve medical decision making by balancing the benefits of correctly treating ill patients and avoiding unnecessary treatment for healthy individuals against the potential harms of missed diagnoses, inappropriate treatments, and the costs and risks associated with testing. We quantify the incremental net benefit (INB) of single and multiple tests by accounting for a patient's pre‐test probability of disease and the associated benefits, harms, and cost of treatment and testing. We decompose the INB into two components: one that captures the value of information provided by the test, independent of the cost and possible harm of testing, and another that accounts for test costs and harm. Next, we examine conjunctive, disjunctive, and majority aggregation functions, demonstrating their application through examples in prostate cancer, colorectal cancer, and stable coronary artery disease diagnostics. Our approach complements traditional threshold and decision‐curve analysis by varying both the pre‐test probability of disease and the cost‐benefit trade‐off of treatment to identify the region over which a given test provides the highest INB. Using empirical test and cost data, we compute decision boundaries to determine when conjunctive, disjunctive, majority, or even single tests are optimal, and, for combinations of tests, in what order they should be administered. In all three application examples, we find that the optimal choice and sequence of tests jointly depend on the probability of disease and the cost‐benefit trade‐off of treatment. An online tool that visualizes the INB for combined tests is available at https://optimal‐testing.streamlit.app/.

## 1 Introduction

The aggregation of results from diagnostic and screening tests helps to improve overall test performance [1, 2, 3, 4, 5, 6, 7, 8, 9]. Different terms are used in the literature to describe various combinations of single tests. For example, the protocol that classifies an individual as diseased if all tests return positive results is referred to as the “all heuristic” [6, 7], “believe‐the‐negative rule” [10], “conjunctive positivity criterion” [3, 11, 12], and “orthogonal testing” [13]. In Boolean algebra, this way of aggregating binary signals corresponds to using the binary AND operator. It implies that once a result is negative, testing stops and the patient remains untreated. Another aggregation method is called “any heuristic” [6, 7] also known as the “believe‐the‐positive rule” [10] or the “disjunctive positivity criterion” [3, 11, 12]. In this protocol, all tests must return negative results to classify an individual as healthy. Therefore, a single positive test is sufficient for a diagnosis, which in turn leads to treatment. In Boolean algebra, this aggregation method corresponds to the binary OR operator.

During the COVID‐19 pandemic, various antigen and antibody tests were developed [14]. Similarly, multiple tests are available in various clinical settings, including diabetes testing [15, 16], medical imaging [17, 18, 19], prostate cancer testing [20], colorectal cancer testing [21], and stable coronary artery disease testing [22].

With multiple tests available, how can one efficiently combine them to maximize their informational value? Although efficient combinations are straightforward for two tests, calculations become increasingly complex as the number of tests increases. In the literature, an algorithm has been proposed to combine test results and identify efficient combinations, using a formulation of a knapsack problem [7]. Another approach derives aggregated sensitivities and specificities from individual tests [23]. While both approaches can identify the receiver operating characteristic (ROC) frontier to combine constituent tests, neither provides a criterion for selecting the optimal combination.

The optimal test along a given ROC curve can be determined by considering the benefits of true positives, the utility loss of false positives, the cost of treatment, and the probability that the patient actually has the condition [12]. This approach assumes that the test characteristics are valid for the target population, which may require periodic re‐evaluation or recalibration. Interestingly, when harm and cost of testing are taken into account, tests that are inefficient from an informational perspective (i.e., tests that are below the ROC curve) could still be optimal. Furthermore, the optimal combination of individual tests will also take into account their optimal ranking order. Tests that are relatively cheap and harmless and that lead to an early termination of testing due to the chosen positivity criterion may be prioritized.

Our work is related to the literature on medical decision‐making under diagnostic risk, which distinguishes between two approaches to the rational use of tests. Pauker and Kassirer (1975, 1980) [24, 25] developed threshold analysis, deriving upper and lower bounds on the pre‐test probability of disease and defining a testing interval based on the cost‐benefit ratio of treatment. Vickers (2008) [26] and Vickers and Elkin (2006) [27], through decision‐curve analysis, focused on establishing lower and upper bounds on the cost‐benefit ratio of treatment to justify the use of a test, given a specific pre‐test probability of disease.

We apply a unified approach, based on the incremental net benefit (INB) of a test, that integrates threshold analysis and decision‐curve analysis, allowing for the simultaneous variation of both the probability of disease and the cost‐benefit ratio of treatment. This incremental approach follows the tradition of the value‐of‐information framework, introduced by Gould (1975) [28], which compares the expected utility of optimal decisions with and without the availability of a test.^1^

In addition to presenting a detailed overview of key mathematical concepts relevant to the analysis of diagnostic tests, we account for different types of harm (e.g., medical risks and economic costs) that influence the optimal order in which a sequence of tests should be administered. Furthermore, our approach complements traditional decision‐curve analysis by varying both the probability of disease and the cost‐benefit trade‐off to identify the region over which a given test provides the highest INB.

To establish criteria for optimally aggregating the test results, the remainder of this paper is organized as follows. The next section provides an overview of key parameters used to mathematically characterize the value of diagnostic information, as well as the benefits and risks associated with specific treatments. Subsequently, we derive the INB for different test combinations and show how the optimal choice and sequence of tests can be framed as a problem of maximizing this function. We then present three applications related to prostate cancer, colorectal cancer, and stable coronary artery disease diagnostics. For all three examples, we identify decision boundaries that determine when different combinations of tests should be used, depending on the cost‐benefit trade‐off of treatment and a patient's probability of disease. Finally, we discuss the findings and conclude the paper. An online tool that we developed to visualize the INB for various combinations of tests and parameters is available at https://optimal‐testing.streamlit.app/.

## 2 The Incremental Net Benefit of a Test

### 2.1 The Treatment Thresholds

We consider a diagnostic risk scenario in which uncertainty affects both the probability of a patient having a disease and the potential benefits and harms of treatment. For an ill patient, a decision maker evaluates the benefit of a treatment

(1) $$b=\lambda q_{g}-c^{\text{Rx}}\;$$

where $q_{g}$ is the gain in quality adjusted life years (QALY), λ is the willingness to pay for a QALY, and $c^{\text{Rx}}$ is the treatment cost.^2^ We use “Rx”, commonly employed in medical contexts to indicate a prescription, to distinguish treatment costs from other costs discussed in subsequent sections.

In contrast, a healthy patient will incur a utility loss

(2) $$l=\lambda q_{l}+c^{\text{Rx}}$$

from treatment, where $q_{l}$ is the loss of QALYs. We now assume that the potential benefits, harms, and costs of treatment vary for each individual patient. Using Equations (1) and (2), we define the cost‐benefit trade‐off of a patient associated with treatment as^3^

(3) $$\rho =\frac{l}{l+b}\;$$

and the uncertainty about the health status is described by the probability of the disease before the test p, which also differs between patients. Although in our later applications, we primarily treat p as a population‐level prevalence dependent on age, it can more generally represent individual‐level risk based on factors such as sex, BMI, and blood pressure. For example, if a regression model provides individualized estimates of p, these can be directly incorporated into our framework.

Faced with a patient, characterized by (p,ρ), the decision maker evaluates the trade‐off between treatment and non‐treatment. The patient's expected utility for treatment is 𝔼[U(p,ρ)]=pb−(1−p)l=b(p−(1−p))ρ/(1−ρ), where we substituted ρ/(1−ρ) for l/b after the second equality. If this quantity is positive, treatment is recommended; otherwise, no treatment is preferable. Setting 𝔼[U(p,ρ)]=0 and solving for p and ρ, respectively, we obtain the two treatment thresholds

(4) $$p^{\text{Rx}}(\rho )=\rho$$

and

(5) $$\rho^{\text{Rx}}(p)=p\;$$

at which the decision maker is indifferent between treatment and no treatment.^4^ A patient should only be treated if $p\geq p^{\text{Rx}}$ or, equivalently, if $\rho \leq \rho^{\text{Rx}}$. Equation (4) defines the treatment threshold $p^{\text{Rx}}$ for a given cost‐benefit trade‐off ρ, while Equation (5) defines the cost‐benefit threshold $\rho^{\text{Rx}}$ for a given probability of disease p.

These thresholds apply in the absence of diagnostic testing. When tests are available, the utility function is adjusted to account for both the benefits of correctly identifying patients who do or do not require treatment and the harms associated with unnecessary or missed treatments. This, in turn, leads to adjusted thresholds, which we examine in the following sections.

### 2.2 The Value of Diagnostic Information

The treatment threshold $p^{\text{Rx}}$ plays a central role in determining the informational value of a test, as it defines the decision maker's choice in the absence of a diagnostic test. For a test with sensitivity Se (true positive rate) and specificity Sp (true negative rate), and the characteristics of the patient summarized by p and ρ, the value of diagnostic information is

(6) $$\begin{array}{ll} & \text{VI}\left(p,\rho ,\text{Se},\text{Sp}\right)\\ & =\left\{\begin{array}{ll} p\;\text{Se}\;b-(1-p)(1-\text{Sp})\;l\;,\; & \text{if}\;0\leq p<p^{\text{Rx}}\\ -\;p\;(1-\text{Se})\;b+(1-p)\;\text{Sp}\;l\;,\; & \text{if}\;p^{\text{Rx}}\leq p\leq 1 \end{array}\right.\\ & =b\left\{\begin{array}{ll} p\;\text{Se}-(1-p)(1-\text{Sp})\;\frac{\rho }{1-\rho }\;,\; & \text{if}\;0\leq p<p^{\text{Rx}}\\ -\;p\;(1-\text{Se})+(1-p)\;\text{Sp}\;\frac{\rho }{1-\rho }\;,\; & \text{if}\;p^{\text{Rx}}\leq p\leq 1\; \end{array}\right. \end{array}$$

The function VI(p,ρ,Se,Sp) is the difference between the expected utility of the treatment decision with and without testing. Without testing, patients with a low probability of disease, p, would remain untreated. In contrast, with testing, patients with true positive results receive treatment and gain utility, while those with false positive results suffer a loss of utility. In expected terms, the utility gain from true positive outcomes is pSeb, while the utility loss from false positive outcomes is (1−p)(1−Sp)l. Without a test, treatment is the preferred choice for patients with a high p. With a test, true negative results avoid the loss of utility associated with unnecessary treatment, providing an expected benefit of (1−p)Spl. However, false negative outcomes prevent the patient from receiving the benefits of treatment, resulting in an expected utility loss of −p(1−Se)b.

In the literature, the value of diagnostic information has been conceptualized in several distinct ways. Peirce (1884) [32], in the context of meteorological forecasts, defined the average profit from predicting a tornado as pSeb−(1−p)(1−Sp)l, where b denotes the monetary profit from correctly predicting a tornado, and l the monetary loss from a false prediction (i.e., an “unfulfilled prediction”). He did not consider scenarios with a high probability of tornadoes in which the default action would be to take precautionary measures.

Pauker and Kassirer (1980) [25], possibly unaware of Peirce's contribution, defined b and l as the utility gain from treating sick patients and the utility loss from treating healthy patients, respectively, and derived the test and the test‐treatment thresholds by setting the expected value of the test equal to the expected value of the reference action (i.e., no treatment for low and treatment for high pre‐test probabilities p) and solving for p. In their analysis, b and l, and therefore ρ, are assumed to be the same for all patients.

Vickers and Elkin (2006) [27], in developing decision‐curve analysis, referenced Peirce's work and defined the net benefit of a test as $p\;\text{Se}\;-(1-p\;)(1-\text{Sp})\;p^{\text{Rx}}/(1-p^{\text{Rx}})$, with $p^{\text{Rx}}$ as the Pauker–Kassirer probability threshold. From Equation (4), we know that $p^{\text{Rx}}=\rho$. Thus, Vickers and Elkin's net‐benefit equation corresponds to the third line of Equation (6) under the assumption b=1.^5^ Like Peirce, Vickers and Elkin focused on low‐probability contexts, but unlike him, their formulation omits the explicit use of b and l.

Decision‐curve analysis can thus be treated within the framework of Equation (6), which does not require the assumption b=1. The value‐of‐information concept is more general, as it jointly accounts for patient heterogeneity with respect to both p and ρ, and encompasses several key approaches discussed in the medical decision‐making literature.^6^

### 2.3 Test Thresholds

A test will come with cost $c^{\text{Dx}}$, and may cause harm to the patient in the case of invasive tests, represented by $\lambda q_{h}^{\text{Dx}}$. The superscript “Dx” is a common abbreviation for diagnosis. Together, the quantities $c^{\text{Dx}}$ and $\lambda q_{h}^{\text{Dx}}$ reflect the loss of utility of the test,

(7) $$l^{\text{Dx}}=\lambda q_{h}^{Dx}+c^{\text{Dx}}\;$$

Accounting for test costs and harms in our characterization of diagnostic tests leads to the concept of the incremental net benefit (INB) of testing, defined as the difference in net benefit between the treatment scenarios with and without testing. That is,

(8) $$\text{INB}(p,\rho )=\text{VI}(p,\rho ,\text{Se},\text{Sp})-l^{\text{Dx}}\;$$

Using the INB, one can formulate the necessary and sufficient conditions for selecting the optimal diagnostic test. Let ℐ={1,2,…,n} be the set of all tests available for the detection of a specific disease. The necessary condition for using test i∈ℐ is ${\text{INB}}_{i}(p,\rho )\geq 0$. The sufficient condition requires ${\text{INB}}_{i}(p,\rho )\geq {\text{INB}}_{j}(p,\rho )$ for all i≠j∈ℐ. Setting Equation (8) equal to zero and solving for p, we obtain the corresponding test and the test‐treatment thresholds [25], given by

(9) $$\begin{array}{ll} {\underline{p}}^{\text{Dx}}(\rho ) & =\frac{(1-\text{Sp})\;l+l^{\text{Dx}}}{(1-\text{Sp})\;l+\text{Se}\;b}\\ & =\frac{\rho \;(1-\text{Sp})+(1-\rho )l^{\text{Dx}}⁄b}{\rho \;(1-\text{Sp})+(1-\rho )\;\text{Se}}\;,\;\text{if}\;p<p^{\text{Rx}}\;\; \end{array}$$

(10) $$\begin{array}{ll} {\bar{p}}^{\text{Dx}}(\rho ) & =\frac{\text{Sp}\;l-l^{\text{Dx}}}{\text{Sp}\;l+(1-\text{Se})\;b}\\ & =\frac{\rho \;\text{Sp}-(1-\rho )l^{\text{Dx}}⁄b}{\rho \;\text{Sp}+(1-\rho )(1-\text{Se})}\;,\;\text{if}\;p\geq p^{\text{Rx}}\; \end{array}$$

Treatment and testing should be withheld if the probability of disease is below the test threshold ${\underline{p}}^{\text{Dx}}(\rho )$. If the probability of disease exceeds the test‐treatment threshold ${\bar{p}}^{\text{Dx}}(\rho )$, treatment should be administered without prior testing. If the probability of disease falls between the two thresholds, the test should be performed. The test interval $\left[{\underline{p}}^{\text{Dx}},{\bar{p}}^{\text{Dx}}\right]$ decreases if a test becomes more costly or more harmful. Starting from the INBs of two tests, i and j≠i, the probability of disease at which the decision maker shifts from preferring test i over test j is

(11) $$p_{ij}^{\text{Dx}}(\rho )=\frac{\rho \;\Delta \text{Sp}\;-\;(1-\rho )\;\Delta l^{\text{Dx}}⁄b}{\rho \;\Delta \text{Sp}\;-(1-\rho )\;\Delta \text{Se}}\;,\;\text{if}\;{\underline{p}}^{\text{Dx}}\leq p\leq {\bar{p}}^{\text{Dx}}\;$$

where $\Delta l^{\text{Dx}}=l_{i}^{\text{Dx}}-l_{j}^{\text{Dx}}$, $\Delta \text{Se}={\text{Se}}_{i}-{\text{Se}}_{j}$, and $\Delta \text{Sp}={\text{Sp}}_{i}-{\text{Sp}}_{j}$.

**FIGURE 1:**
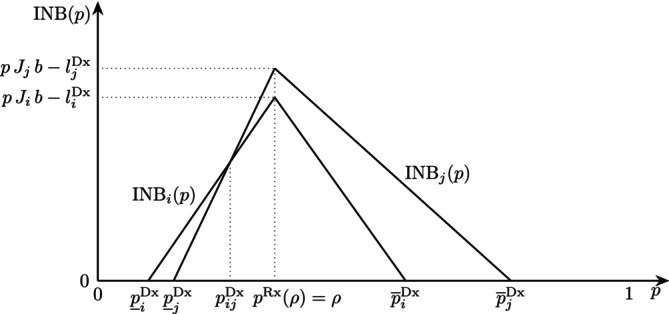
The incremental net benefits ${\text{INB}}_{i}(p)$ and ${\text{INB}}_{j}(p)$ of two tests as a function of the probability of disease p, given ρ. The test and test‐treatment thresholds are ${\underline{p}}_{i}^{\text{Dx}},{\underline{p}}_{j}^{\text{Dx}}$ and ${\bar{p}}_{i}^{\text{Dx}},{\bar{p}}_{j}^{\text{Dx}}$, respectively. At $p_{ij}^{\text{Dx}}$, the decision maker shifts from preferring test i over test j.

In Figure 1, we show the INB of two tests as a function of the probability of disease p. The INB is linear in p and reaches its maximum value, $p\;J\;b-l^{\text{Dx}}$, at ρ, where J=Se−(1−Sp) is the Youden index [34]. The test interval is determined by the minimum test thresholds and the maximum test‐treatment thresholds. In the scenario shown in Figure 1, patients with $p<{\underline{p}}_{i}^{\text{Dx}}$ or $p\geq {\bar{p}}_{j}^{\text{Dx}}$ should not be tested. The former should not be treated and the latter undergo direct treatment. For ${\underline{p}}_{i}^{\text{Dx}}\leq p\leq p_{ij}^{\text{Dx}}$, test i is recommended, and for $p_{ij}^{\text{Dx}}\leq p\leq {\bar{p}}_{j}^{\text{Dx}}$, test j is preferred.

Rather than expressing the thresholds in Equations ((9), (10), (11)) as a function of a patient's cost‐benefit trade‐off ρ, we can alternatively derive thresholds for ρ, given a patient's probability of disease p [27]. Setting Equation (8) equal to zero and solving for ρ yields lower and upper bounds

(12) $$\begin{array}{llll} {\underline{\rho }}^{\text{Dx}}(p) & =\frac{p\;(1-\text{Se})+l^{\text{Dx}}⁄b}{p\;(1-\text{Se})+(1-p)\;\text{Sp}+l^{\text{Dx}}⁄b},\;\text{if}\;p^{\text{Rx}}\leq p\;, & & \\ {\bar{\rho }}^{\text{Dx}}(p) & =\frac{p\;\text{Se}-l^{\text{Dx}}⁄b}{p\;\text{Se}+(1-p)(1-\text{Sp})-l^{\text{Dx}}⁄b},\;\text{if}\;p^{\text{Rx}}>p\; & & \end{array}$$

where a test with sensitivity Se, specificity Sp, and utility loss $l^{\text{Dx}}$ can be used.

The width of the test interval $\left[{\underline{\rho }}^{\text{Dx}},{\bar{\rho }}^{\text{Dx}}\right]$ increases with $l^{\text{Dx}}$. When the test is cost‐free and does not cause harm, the upper threshold corresponds to the positive predictive value, and the lower threshold corresponds to 1 minus the negative predictive value.

The treatment threshold at which the decision maker shifts from preferring test i over test j is

(13) $$\rho_{ij}^{\text{Dx}}(p)=\frac{p\;\Delta \text{Se}-\Delta l^{\text{Dx}}⁄b}{p\;\Delta \text{Se}-(1-p)\;\Delta \text{Sp}-\Delta l^{\text{Dx}}⁄b}\;$$

**FIGURE 2:**
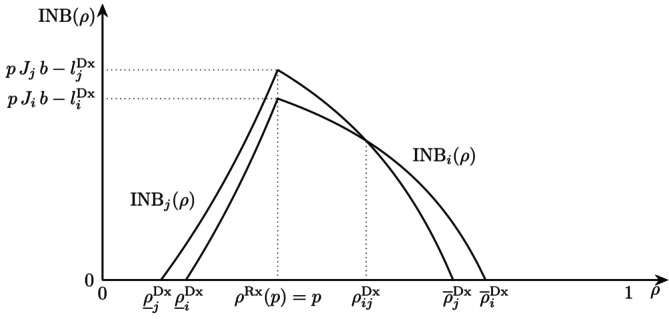
The incremental net benefits ${\text{INB}}_{i}(\rho )$ and ${\text{INB}}_{j}(\rho )$ of two tests as a function of ρ. The lower and upper threshold where testing is indicated are ${\underline{\rho }}_{i}^{\text{Dx}},{\underline{\rho }}_{j}^{\text{Dx}}$ and ${\bar{\rho }}_{i}^{\text{Dx}},{\bar{\rho }}_{j}^{\text{Dx}}$, respectively. The probability of disease is p. At $\rho_{ij}^{\text{Dx}}$, the decision maker shifts from preferring test i over test j.

In Figure 2, we show the INB of two tests as a function of ρ. The INB is convex for ρ<p and concave for ρ>p. Given the probability of disease p, and the characteristics of the tests, including their costs, a testing range $\left[{\underline{\rho }}^{\text{Dx}},{\bar{\rho }}^{\text{Dx}}\right]$ is defined. Patients for which $\rho <{\underline{\rho }}_{j}^{\text{Dx}}$ should be treated without prior testing, while for patients with $\rho \geq {\bar{\rho }}_{j}^{\text{Dx}}$, neither testing nor treatment is indicated. Patients in the range ${\underline{\rho }}_{j}^{\text{Dx}}\leq \rho <\rho_{ji}^{\text{Dx}}$ should undergo test j, and those in the range $\rho_{ji}^{\text{Dx}}\leq \rho <{\bar{\rho }}_{i}^{\text{Dx}}$ should receive test i.

## 3 Aggregating Test Results

With multiple tests available, the decision maker must determine how to aggregate the test results, choose a positivity criterion, and decide the order in which the tests will be conducted. In a conjunctive approach (using the AND operator in Boolean algebra), each subsequent test is performed if and only if the previous test is positive, and testing stops immediately upon a negative result. In a disjunctive approach (using the OR operator), additional tests are conducted if and only if the previous test is negative, and the test stops as soon as a positive result is obtained.

We use the notation x∧y and x∨y to denote the Boolean functions xANDy and xORy, respectively. When two tests are administered under either approach, the following interpretations apply:

- 1∧2: Do test 1; if positive, do test 2. Treat if the patient is positive for both tests.
- 1∨2: Do test 1; if negative, do test 2. Treat if the patient is positive for either test.
- 2∧1: Do test 2; if positive, do test 1. Treat if the patient is positive for both tests.
- 2∨1: Do test 2; if negative, do test 1. Treat if the patient is positive for either test.

When more than two tests are available, the results can be aggregated using combinations of the AND and OR functions. In particular, a majority criterion, in which treatment is initiated if most tests are positive, can be viewed as a specific combination of these operators. The cost and harm of each individual test will play a key role in determining the specific sequence of tests.

In deriving our results, we assume that the outcomes of different tests are conditionally independent, given the state of the disease. This assumption is commonly used in the medical decision‐making literature as it simplifies the mathematical analysis of aggregated test results. In addition, manufacturers usually report performance measures for individual tests without specifying potential dependencies between them. However, in practice, test results may be correlated.

### 3.1 Two Tests

The incremental net benefit of a sequence consisting of two tests, starting with test 1 and using the AND operator, is

(14) $$\begin{array}{ll} {\text{INB}}_{1\wedge 2}\left(p,\rho \right) & =\text{VI}\left(p,\rho ,{\text{Se}}_{1\wedge 2},{\text{Sp}}_{1\wedge 2}\right)\\ & \;-l_{1}^{\text{Dx}}-\left(p\;{\text{Se}}_{1}+\left(1-p\right)\left(1-{\text{Sp}}_{1}\right)\right)\;l_{2}^{\text{Dx}}\; \end{array}$$

where ${\text{Se}}_{1\wedge 2}=\prod_{i=1}^{2}{\text{Se}}_{i}$ and ${\text{Sp}}_{1\wedge 2}=1-\prod_{i=1}^{2}\left(1-{\text{Sp}}_{i}\right)$. The sequence of tests (i.e., whether to start with test 1 or test 2) does not affect the value of diagnostic information; it only changes the expected testing cost. As $p\;{\text{Se}}_{1}+(1-p)\;(1-{\text{Sp}}_{1})$ is the probability of a positive test result from test 1, test 1 has an advantage over test 2 not only if its cost and potential harm are lower but also if it is expected to produce fewer (true and false) positive results. This is because fewer positive outcomes make it less likely that test 2 will be needed.

The incremental net benefit associated with initiating the test sequence with test 1 and applying the OR operator is

(15) $$\begin{array}{ll} {\text{INB}}_{1\vee 2}\left(p,\rho \right) & =\text{VI}\left(p,\rho ,{\text{Se}}_{1\vee 2},{\text{Sp}}_{1\vee 2}\right)-l_{1}^{\text{Dx}}\\ & \;-\left(p\;\left(1-{\text{Se}}_{1}\right)+\left(1-p\right)\;{\text{Sp}}_{1}\right)\;l_{2}^{\text{Dx}}\; \end{array}$$

where ${\text{Se}}_{1\vee 2}=1-\prod_{i=1}^{2}\left(1-{\text{Se}}_{i}\right)$ and ${\text{Sp}}_{1\vee 2}=\prod_{i=1}^{2}{\text{Sp}}_{i}$. Again, the test sequence does not affect the value of diagnostic information. The expected cost and harm of test 2 depend on $p\;(1-{\text{Se}}_{1})+(1-p)\;{\text{Sp}}_{1}$, the probability of a negative result from test 1.

### 3.2 Three Tests

For three tests, the incremental net benefit associated with the AND operator is

(16) $$\begin{array}{ll} {\text{INB}}_{1\wedge 2\wedge 3}\left(p,\rho \right)= & \text{VI}\left(p,\rho ,{\text{Se}}_{1\wedge 2\wedge 3},{\text{Sp}}_{1\wedge 2\wedge 3}\right)-l_{1}^{\text{Dx}}\\ & -\left(p\;{\text{Se}}_{1}+\left(1-p\right)\left(1-{\text{Sp}}_{1}\right)\right)\;l_{2}^{\text{Dx}}\\ & -\left(p\;{\text{Se}}_{1\wedge 2}+\left(1-p\right)\left(1-{\text{Sp}}_{1\wedge 2}\right)\right)\;l_{3}^{\text{Dx}}\; \end{array}$$

where ${\text{Se}}_{1\wedge 2\wedge 3}=\prod_{i=1}^{3}{\text{Se}}_{i}$ and ${\text{Sp}}_{1\wedge 2\wedge 3}=1-\prod_{i=1}^{3}\left(1-{\text{Sp}}_{i}\right)$. Building on the INB from the two‐test case [see Equation (14)], we incorporate the term $p\;{\text{Se}}_{1\wedge 2}+\left(1-p\right)\;\left(1-{\text{Sp}}_{1\wedge 2}\right)=p\;{\text{Se}}_{1}{\text{Se}}_{2}+\left(1-p\right)\;\left(1-{\text{Sp}}_{1}\right)\;\left(1-{\text{Sp}}_{2}\right)$. This term accounts for the probability of a positive outcome after two tests, which leads to the use of the third test. As in the two‐test examples, the specific test sequence does not affect the value of information; it only influences the expected cost and harm of testing.

For the OR operator, we have

(17) $$\begin{array}{ll} {\text{INB}}_{1\vee 2\vee 3}\left(p,\rho \right)= & \text{VI}\left(p,\rho ,{\text{Se}}_{1\vee 2\vee 3},{\text{Sp}}_{1\vee 2\vee 3}\right)-l_{1}^{\text{Dx}}\\ & -\left(p\;\left(1-{\text{Se}}_{1}\right)+\left(1-p\right)\;{\text{Sp}}_{1}\right)\;l_{2}^{\text{Dx}}\\ & -\left(p\;\left(1-{\text{Se}}_{1\vee 2}\right)+\left(1-p\right)\;{\text{Sp}}_{1\vee 2}\right)\;l_{3}^{\text{Dx}}\; \end{array}$$

where ${\text{Se}}_{1\vee 2\vee 3}=1-\prod_{i=1}^{3}(1-{\text{Se}}_{i})$ and ${\text{Sp}}_{1\vee 2\vee 3}=\prod_{i=1}^{3}{\text{Sp}}_{i}$. The probability of a negative outcome after two tests, which necessitates the use of a third test, is $p\;\left(1-{\text{Se}}_{1\vee 2}\right)+\left(1-p\right)\;{\text{Sp}}_{1\vee 2}=p\;(1-{\text{Se}}_{1})\;(1-{\text{Se}}_{2})+(1-p)\;{\text{Sp}}_{1}\;{\text{Sp}}_{2})$.

With three tests, a test protocol based on the majority criterion offers another combinatorial option. If two tests yield positive outcomes, the decision maker would choose treatment, whereas two negative outcomes would lead to no treatment. The third test is required only when the first two tests produce conflicting results. This approach results in the incremental net benefit

(18) $$\begin{array}{ll} {\text{INB}}_{M(1,2,3)}\left(p,\rho \right) & =\text{VI}\left(p,\rho ,{\text{Se}}_{M(1,2,3)},{\text{Sp}}_{M(1,2,3)}\right)-l_{1}^{\text{Dx}}-l_{2}^{\text{Dx}}\\ & \;-\left[p\;\left({\text{Se}}_{1}\;(1-{\text{Se}}_{2})+(1-{\text{Se}}_{1})\;{\text{Se}}_{2}\right)\right.\\ & \;+\left.(1-p)\left({\text{Sp}}_{1}\;(1-{\text{Sp}}_{2})+(1-{\text{Sp}}_{1})\;{\text{Sp}}_{2}\right)\right]\;l_{3}^{\text{Dx}}\; \end{array}$$

where

(19) $${\text{Se}}_{M(1,2,3)}={\text{Se}}_{1}{\text{Se}}_{2}+{\text{Se}}_{1}{\text{Se}}_{3}+{\text{Se}}_{2}{\text{Se}}_{3}-2{\text{Se}}_{1}{\text{Se}}_{2}{\text{Se}}_{3}$$

and

(20) $${\text{Sp}}_{M(1,2,3)}={\text{Sp}}_{1}{\text{Sp}}_{2}+{\text{Sp}}_{1}{\text{Sp}}_{3}+{\text{Sp}}_{2}{\text{Sp}}_{3}-2{\text{Sp}}_{1}{\text{Sp}}_{2}{\text{Sp}}_{3}\;$$

We use the notation M(1,2,3) to denote a majority function in which tests 1, 2, and 3 are applied sequentially in that order.

### 3.3 AND and OR Aggregation of the Results of n Tests

Generalizing the previous equations to cases with n>3 tests is straightforward. Adding another test affects the overall informational value of the protocol, increases the expected cost—including a potential harm in case of an invasive test—and may trigger further testing, depending on the chosen positivity criterion. The incremental net benefit of a combined n‐test, when using the AND operator, is

(21) $$\begin{array}{ll} {\text{INB}}_{1\wedge \cdots \wedge n}\left(p,\rho \right)= & \text{VI}\left(p,\rho ,{\text{Se}}_{1\wedge \cdots \wedge n},{\text{Sp}}_{1\wedge \cdots \wedge n}\right)-l_{1}^{\text{Dx}}\\ & -\sum_{i=2}^{n-1}\left[p\;(1-{\text{Se}}_{1\wedge \cdots \wedge i})+(1-p)\;{\text{Sp}}_{1\wedge \cdots \wedge i}\right]\;l_{i}^{\text{Dx}}\; \end{array}$$

where

(22) $${\text{Se}}_{1\wedge \cdots \wedge n}=\prod_{i=1}^{n}{\text{Se}}_{i}\;\text{and}\;{\text{Sp}}_{1\wedge \cdots \wedge n}=1-\prod_{i=1}^{n}(1-{\text{Sp}}_{i})$$

With the AND operator, overall sensitivity decreases, while overall specificity increases with n. In environments where the probability of disease is low, increasing the number of tests is appealing. A high specificity decreases the expected number of false positives, which is advantageous both from the informational and the cost perspectives. At the same time, applying one more test always implies an additional cost.

For the OR operator, we have

(23) $$\begin{array}{ll} {\text{INB}}_{1\vee \cdots \vee n}\left(p,\rho \right)= & \text{VI}\left(p,\rho ,{\text{Se}}_{1\vee \cdots \vee n},{\text{Sp}}_{1\vee \cdots \vee n}\right)-l_{1}^{\text{Dx}}\\ & -\overset{n-1}{\underset{i=2}{\sum }}\left[p(1-{\text{Se}}_{1\vee \cdots \vee i})+(1-p){\text{Sp}}_{1\vee \cdots \vee i}\right]\;l_{i}^{\text{Dx}} \end{array}$$

where

(24) $${\text{Se}}_{1\vee \cdots \vee n}=1-\overset{n}{\underset{i=1}{\prod }}(1-{\text{Se}}_{i})\;\text{and}\;{\text{Sp}}_{1\vee \cdots \vee n}=\overset{n}{\underset{i=1}{\prod }}{\text{Sp}}_{i}$$

With the OR operator, overall sensitivity increases, while overall specificity decreases with n. If the probability of illness is high, decision makers will be inclined to increase the number of tests because a high sensitivity decreases the expected number of false negatives which is warranted both from the informational and the cost perspectives.

As shown in Equations ((14), (15), (16), (17), (18)), the value of diagnostic information does not depend on the order in which the n tests are conducted. Low‐cost tests, when performed earlier in the sequence, are associated with a lower INB. Under the AND operator, tests that decrease the probability of positive outcomes are preferred due to their lower INB. In contrast, under the OR operator, tests that reduce the probability of negative outcomes are more likely to be prioritized earlier in the sequence.

For test strategies using the majority rule, we do not present the INB, overall sensitivity, or specificity when n>3 and odd, as the corresponding mathematical expressions become very lengthy and their derivation is considerably more complex than for the AND and OR functions.

### 3.4 Determining the Optimal Test

While the value of diagnostic information remains unaffected by the order of tests in a given sequence, the incremental net benefit can vary depending on that order. To identify the optimal single or combined test, we proceed as follows. We use 𝒮 to denote a set of available single and combined tests. Given a patient's pre‐test probability of disease p and cost‐benefit trade‐off ρ, the decision maker will select the test

(25) $$k^{*}=\underset{k\in 𝒮}{\text{argmax}}{\text{INB}}_{k}(p,\rho )\;$$

For the region in the (p,ρ) space where testing is indicated, the decision maker's choice can also be described using the thresholds defined in Section 2. For the set 𝒮 of available single and combined tests, we have

(26) $${\underline{p}}_{\min}^{\text{Dx}}(\rho )=\underset{k\in 𝒮}{\min}\left({\underline{p}}_{k}^{\text{Dx}}(\rho )\right)={\bar{\rho }}_{\max}^{\text{Dx},-1}(p)$$

and

(27) $${\bar{p}}_{\max}^{\text{Dx}}(\rho )=\max_{k\in 𝒮}\left({\bar{p}}_{k}^{\text{Dx}}(\rho )\right)={\underline{\rho }}_{\min}^{\text{Dx},-1}(p)\;$$

where ${\underline{\rho }}_{\min}^{\text{Dx}}(p)=\min_{k\in 𝒮}({\underline{\rho }}_{k}^{\text{Dx}}(p))$ and ${\bar{\rho }}_{\max}^{\text{Dx}}(p)=\max_{k\in 𝒮}({\bar{\rho }}_{k}^{\text{Dx}}(p))$. We use the notation ${\bar{\rho }}_{\max}^{\text{Dx},-1}(p)$ and ${\underline{\rho }}_{\min}^{\text{Dx},-1}(p)$ to indicate the inverse of ${\underline{p}}_{\min}^{\text{Dx}}(\rho )$ and ${\bar{p}}_{\max}^{\text{Dx}}(\rho )$, respectively.

This brings us to the following decision rules for a patient characterized by (p,ρ):

- Do not test or treat if $p<{\underline{p}}_{\min}^{\text{Dx}}(\rho )$ or, equivalently, $\rho >{\underline{\rho }}_{\max}^{\text{Dx}}(p)$
- Test if ${\underline{p}}_{\min}^{\text{Dx}}(\rho )\leq p\leq {\bar{p}}_{\max}^{\text{Dx}}(\rho )$ or, equivalently, ${\underline{\rho }}_{\min}^{\text{Dx}}(p)\leq \rho \leq {\bar{\rho }}_{\max}^{\text{Dx}}(p)$
- Treat without testing if $p>{\bar{p}}_{\max}^{\text{Dx}}(\rho )$ or, equivalently, $\rho <{\underline{\rho }}_{\min}^{\text{Dx}}(p)$

Within the region where testing is indicated, the optimal transition threshold can be determined by comparing all pairs $p_{ij}^{\text{Dx}}(\rho )$ and $\rho_{ij}^{\text{Dx}}(p)$. However, this approach requires quadratic memory and runtime, as every pair of tests must be evaluated, making it computationally complex. A more efficient method, linear in the number of tests, involves computing the envelope of ${\text{INB}}_{k}(p,\rho )$ and directly determining the optimal single or multiple test and corresponding transition thresholds between tests using Equation (25).

The optimal order of combined tests depends only on the utility losses of the individual tests involved. For instance, in the AND combination with two tests, starting with test 1 implies a direct utility loss of $l_{1}^{\text{Dx}}$, but may save test 2's utility loss in the case of a negative test outcome. The total savings amount to $\left(p\;\left(1-{\text{Se}}_{1}\right)+\left(1-p\right)\;{\text{Sp}}_{1}\right)l_{2}^{\text{Dx}}$, where $p\;\left(1-{\text{Se}}_{1}\right)+\left(1-p\right)\;{\text{Sp}}_{1}$ is the probability of a negative test outcome with test 1. The comparison between direct utility losses and the expected utility savings due to a negative test outcome determines the optimal test order under the AND function. Under the OR function, savings occur due to a positive test outcome, which results in treatment without further testing.

## 4 Applications

We now turn to three applications to illustrate how the choice of the optimal test protocol varies with both the probability of disease p and the cost‐benefit trade‐off of treatment ρ. In all three applications, we identify the optimal single test or combination of tests by evaluating all possible sequences and selecting the one that maximizes the incremental net benefit [see Equation (25)]. We also include ROC plots, which show tests in (sensitivity,1−specificity) space, to help identify efficient tests, defined as those that lie on the ROC frontier. While these tests maximize informational value by offering the best trade‐off between sensitivity and specificity, they may not be optimal overall once test costs and potential harms are taken into account.

The first two examples focus on prostate cancer and colorectal cancer diagnostics, two diseases which exhibit a low prevalence. The third example considers stable coronary artery disease, a condition with relatively high prevalence in certain population groups. In all three cases, up to three tests can be combined using the AND, OR, and majority functions.

### 4.1 Prostate Cancer Diagnostics

Prostate‐specific antigen (PSA) levels in the blood are used to identify men with prostate cancer. A cutoff of 20% for free‐to‐total PSA (FT) is applied to define a positive test result. Alternatively, or as a complement, human kallikrein 2 (hK2) can be used, with a cutoff set at 0.075 ng/mL. For these cutoffs, Vickers et al. (2013) report ${\text{Se}}_{\text{FT}}=0.91$ and ${\text{Sp}}_{\text{FT}}=0.40$ for FT, and ${\text{Se}}_{\mathrm{hK}2}=0.51$ and ${\text{Sp}}_{\mathrm{hK}2}=0.78$ for hK2 [20]. Although the Youden index differs only slightly between the two tests ($J_{\text{FT}}=0.31$ versus $J_{\mathrm{hK}2}=0.29$), FT clearly dominates HK in terms of the positive likelihood ratio (${\text{LR}}_{\text{FT}}^{+}=2.32$ versus ${\text{LR}}_{\mathrm{hK}2}^{+}=1.52$). However, FT is inferior to HK with respect to the negative likelihood ratio (${\text{LR}}_{\text{FT}}^{-}=0.66$ versus ${\text{LR}}_{\mathrm{hK}2}^{-}=0.43$).^7^

**FIGURE 3:**
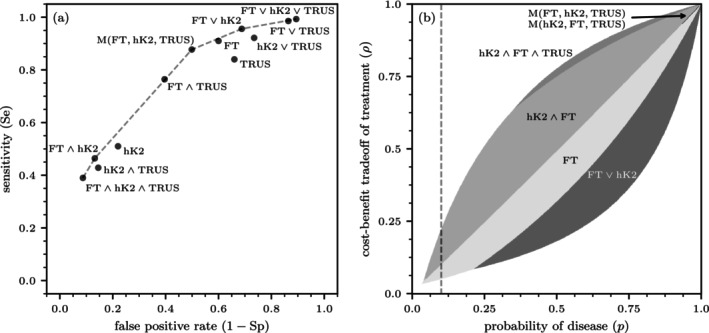
Prostate cancer diagnostics. (a) The ROC curve for prostate cancer testing. (b) Regions within the (p,ρ) unit square where different testing protocols are optimal. In the white regions, the INB for all available test sequences is negative, indicating that no testing should be performed. The sequence of tests does not influence the sensitivity and specificity values shown in panel (a). However, it is crucial in determining the optimal testing protocols illustrated in panel (b). The dashed grey line indicates a typical upper limit of prostate‐cancer prevalence in the general population. Because the order of the first two tests, FT and hK2, does not affect the outcome under the majority function M(·), the aggregations M(FT,hK2,TRUS) and M(hK2,FT,TRUS) are equivalent in terms of their incremental net benefits. Since TRUS is an invasive test with substantially higher costs and associated harms compared to FT and hK2, it is administered last.

A third option for prostate cancer testing is transrectal ultrasound (TRUS). For a cutoff of TRUS volume equal to 50 ${\text{cm}}^{3}$, Vickers et al. (2013) report ${\text{Se}}_{\text{TRUS}}=0.84$ and ${\text{Sp}}_{\text{TRUS}}=0.34$. Single tests and combined tests with varying sequences and positivity criteria result in 33 different test protocols (see Table 1). Since the sequence of tests does not affect the resulting sensitivity and specificity, these protocols produce 12 distinct pairs of sensitivity and specificity. From an information‐theoretic perspective, nine of these test protocols are efficient, as they are part of the ROC frontier (see Figure 3a).^8^ The individual tests FT, hK2, and TRUS, as well as the combined tests hK2∧TRUS, hK2∨TRUS, and FT∨TRUS, are not efficient as they are all weakly dominated by combinations of neighboring tests.

**TABLE 1:** Sensitivity and specificity of single and combined tests, as well as their optimal intervals, for prostate cancer diagnosis. We assume that $l_{\text{TRUS}}^{\text{Dx}}/b=0.1$ and $l_{\text{FT}}^{\text{Dx}}/b=l_{\mathrm{hK}2}^{\text{Dx}}/b=0.01$.

Test	Se	Sp	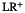	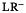	Efficient test	Optimal test interval	
						$p^{\text{Dx}}(\rho =0.26)$	$\rho^{\text{Dx}}(p=0.26)$
Single tests							
FT	0.91	0.40	1.52	0.23	no	[0.27, 0.44]	[0.12, 0.26]
hK2	0.51	0.78	2.32	0.63	no		
TRUS	0.84	0.34	1.27	0.47	no		
Combined tests							
AND (n=2)							
FT, hK2	0.46	0.87	3.54	0.62	yes	[0.12, 0.27]	[0.26, 0.53]
hK2, FT	0.46	0.87	3.54	0.62	yes		
FT, TRUS	0.76	0.60	1.90	0.40	yes		
TRUS, FT	0.76	0.60	1.90	0.40	yes		
hK2, TRUS	0.43	0.85	2.87	0.67	no		
TRUS, hK2	0.43	0.85	2.87	0.67	no		
AND (n=3)	0.39	0.91	4.33	0.67	yes		
FT, hK2, TRUS; FT, TRUS, hK2; hK2, FT, TRUS; hK2, TRUS, FT; TRUS, FT, hK2; TRUS, hK2, FT							
OR (n=2)							
FT, hK2	0.96	0.31	1.39	0.13	yes	[0.44, 0.63]	[0.1, 0.12]
hK2, FT	0.96	0.31	1.39	0.13	yes		
FT, TRUS	0.99	0.14	1.15	0.07	no		
TRUS, FT	0.99	0.14	1.15	0.07	no		
hK2, TRUS	0.92	0.27	1.26	0.30	no		
TRUS, hK2	0.92	0.27	1.26	0.30	no		
OR (n=3)	0.99	0.11	1.11	0.09	yes		
FT, hK2, TRUS; FT, TRUS, hK2; hK2, FT, TRUS; hK2, TRUS, FT; TRUS, FT, hK2; TRUS, hK2, FT							
Majority (n=3)	0.88	0.55	1.96	0.22	yes		
FT, hK2, TRUS; FT, TRUS, hK2; hK2, FT, TRUS; hK2, TRUS, FT; TRUS, FT, hK2; TRUS, hK2, FT							

TRUS involves inserting a probe into a patient's rectum, which is uncomfortable for the patient and time‐consuming for the physician. Vickers et al. (2013) quote an urologist who stated that he would perform no more than 10 ultrasound tests to detect cancer if the ultrasound was a perfect test. Assuming that this urologist anticipated the benefits, harm, and cost of a biopsy, as well as of cancer treatment for true positives, we set $b=10l^{\text{Dx}}$. For their study on biopsy outcomes, Vickers et al. (2013) report that 26% of patients tested positive for cancer.

For $\rho^{\text{Rx}}=p$, the informational value of a test is maximized. For $\rho^{\text{Rx}}=0.26$, which corresponds to a benefit‐cost ratio b/l of 2.85 in treatment, the overall test range for the pre‐test probability of disease is [0.12,0.63] (Table 1). The first optimal test within this range is the combined test hK2∧FT, with ${\underline{p}}_{\mathrm{hK}2\wedge \text{FT}}^{\text{Dx}}=0.12$. Among all single and double tests, it has the highest positive likelihood ratio. For the combined test hK2∧FT, hK2 is performed first because its higher specificity compared to FT reduces the probability of positive test outcomes and consequently decreases the probability that FT will be conducted. At $p_{\mathrm{hK}2\wedge \text{FT},\text{FT}}^{\text{Dx}}=0.27$ (above the treatment threshold), the single FT test begins to offer a greater incremental net benefit than hK2∧FT. Notice that the single FT test has a very low negative likelihood ratio. At $p_{\text{FT},\text{FT}\vee \mathrm{hK}2}^{\text{Dx}}=0.44$, the combined test FT∨hK2, which has the lowest negative likelihood ratio, becomes the optimal testing protocol. The first test in this sequence is FT, which, due to its high sensitivity, reduces the probability of both negative test outcomes and the need for the second test. The test range ends at ${\bar{p}}_{\text{FT}\vee \mathrm{hK}2}^{\text{Dx}}=0.63$.

With the probability of disease fixed at p=0.26 and the cost‐benefit trade‐off ρ varying, the range where testing is indicated is [0.11,0.53]. The lower bound is reached by FT∨hK2, and the upper bound by hK2∧FT. This corresponds to an interval of [8.09,0.89] for b/l where testing is justified. At $\rho_{\text{FT}\vee \mathrm{hK}2,\text{FT}}^{\text{Dx}}=0.12$, the single test FT becomes optimal. Then, at $\rho_{\text{FT},\mathrm{hK}2\wedge \text{FT}}^{\text{Dx}}=0.26$ and for higher values of ρ, the conjunctively combined test hK2∧FT is indicated. The corresponding benefit‐cost ratio for FT and hK2∧FT is 7.33 and 2.85, respectively.

Figure 3b shows the regions within the (p,ρ) unit square where different testing protocols are optimal. The protocols shown correspond to the optimal single test or combination of tests, determined by evaluating all possible test sequences and selecting the one that maximizes the incremental net benefit [see Equation (25)]. Unlike the ROC curve in Figure 3a, which is based solely on the sensitivity and specificity of the tests and is therefore not affected by the order of the tests, the results in Figure 3b depend on the sequence of tests, as they account for both costs and associated harms.

Interestingly, for p>0.3 and ρ>0.65, the conjunctive triple test hK2∧FT∧TRUS can be the optimal choice. However, the range of (p,ρ) combinations, where this is the case, is very narrow. The majority rule becomes a viable option only when p and ρ are around 0.9, where the benefit‐cost ratio of treatment is 9.

Vickers et al. (2013) emphasize the importance of assessing the patient's treatment preferences, which can be determined through a shared decision‐making process [20]. They suggest that the typical ρ for prostate cancer biopsy is 20%, corresponding to a benefit‐cost ratio of 4. As shown in Figure 3b, this threshold roughly translates to a testing interval for p between 0.1 and 0.5.

For Germany, the official 10‐year probabilities of developing prostate cancer for men at various ages are: below 0.1% for those under 35 years, 0.4% at 45 years, 2.5% at 55 years, 6.2% at 65 years, and 6.7% at 75 years [35]. These probabilities are all below the minimum test threshold, indicating that men should not undergo single or combined tests for prostate cancer. Testing would only be reasonable for men over 65 years old if the benefit‐cost ratio for biopsies exceeded 10. At this threshold, the combined test hK2∧FT would be the preferred testing protocol due to its high positive likelihood ratio and low expected testing costs. A benefit‐cost ratio of at least 24 for the biopsy followed cancer treatment would be required to justify using the single FT test.

### 4.2 Colorectal Cancer Diagnostics

In Germany, similar to other industrialized countries, the lifetime risk of developing colorectal cancer is approximately 1 in 25. Below age 65, the incidence rate is under 1%, but it increases to about 2% by age 80 [36]. Many countries have implemented screening programs to detect colorectal cancer in the population. Several diagnostic options are available. The fecal immunochemical test (FIT) uses antibodies to specifically detect hemoglobin protein. Multitarget stool DNA testing (MTsDNA) identifies both hemoglobin and certain DNA biomarkers. Additionally, a colonoscopy examines the rectum, the sigma, and the entire colon using a flexible, lighted tube called a colonoscope. This device is equipped with a lens for viewing and a tool for tissue removal. While this invasive test is effective, it carries a perforation rate of 0.04% and, in the event of endoscopic perforation, a mortality rate of 7.5% [21]. With an assumed residual life expectancy of 15 years, the potential harm to the patient is equivalent to a loss of 0.0006 life years. Additional test characteristics, such as sensitivities and specificities, are summarized in Table 2, based on data from Pickhardt et al. (2003) and Ladabaum & Mannalithara (2016) [21, 37]. Furthermore, treatment is characterized by $c^{\text{Rx}}=\text{USD}\;75\;000$, λ=USD100000, $q_{g}=1.5$, and thus $b=\lambda q_{g}-c^{\text{Rx}}=\text{USD}\;75\;000$.

**TABLE 2:** Characteristics of FIT, MTsDNA, and colonoscopy for colorectal cancer diagnosis.

	FIT	MTsDNA	Colonoscopy
Se	0.733	0.933	0.887
Sp	0.964	0.898	0.796
$c^{\text{Dx}}$	USD 19	USD 649	USD 1400
$q_{h}^{\text{Dx}}$			$6\times 10^{-4}$
$c^{\text{Dx}}/b$	$2.53\times 10^{-4}$	$8.65\times 10^{-3}$	
$l^{\text{Dx}}/b$			$1.97\times 10^{-2}$

**FIGURE 4:**
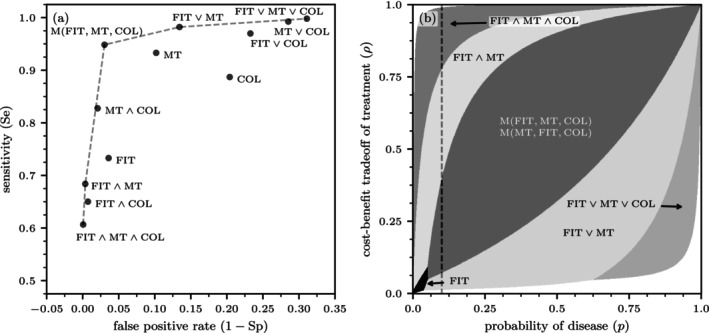
Colorectal cancer diagnostics. (a) The ROC curve for colorectal cancer testing. (b) Regions within the (p,ρ) unit square where different testing protocols are optimal. In the white regions, the INB for all available test sequences is negative, indicating that no testing should be performed. The sequence of tests does not influence the sensitivity and specificity values shown in panel (a). However, it is crucial in determining the optimal testing protocols illustrated in panel (b). The dashed grey line indicates a typical upper limit of colorectal‐cancer prevalence in the general population. Because the order of the first two tests, FIT and MT, does not affect the outcome under the majority function M(·), the aggregations M(FIT,MT,COL) and M(MT,FIT,COL) are equivalent in terms of their incremental net benefits. Since COL is an invasive test with substantially higher costs and associated harms compared to FIT and MT, it is administered last.

Figure 4a shows the ROC curve for colorectal cancer diagnostics. Similar to the previous example, for prostate cancer diagnostics, out of 33 combinations, 12 tests have distinct sensitivities and specificities. Five of these tests form the ROC frontier. None of the single tests belong to the frontier. Remarkably, the majority test protocol, with ${\text{Se}}_{M(\text{FIT},\text{MT},\text{COL})}=0.95$ and ${\text{Sp}}_{M(\text{FIT},\text{MT},\text{COL})}=0.98$, is very close to the maximum values of ${\text{Se}}_{\text{FIT}\vee \text{MT}\vee \text{COL}}=0.99$ and ${\text{Sp}}_{\text{FIT}\wedge \text{MT}\wedge \text{COL}}=0.99$.

Figure 4b shows the different test regions in the (p,ρ) space. Compared to the prostate cancer case, the overall area in which testing is indicated is significantly larger, primarily due to the higher accuracy of the tests for colorectal cancer. The single FIT test, which is not part of the ROC frontier, is the optimal test for low values of p and ρ. The combined test FIT∨MT is optimal for slightly larger values of p. For p>0.05 and sufficiently large values of ρ, tests with majority aggregation function are optimal, provided that COL is used as the last test in the sequence. Whether to begin with FIT or MT makes no difference.

Given the low pre‐test probability of colorectal cancer, only FIT and conjunctively combined tests with high specificities (${\text{Sp}}_{\text{FIT}\wedge \text{MT}\wedge \text{COL}}=0.999$ and ${\text{Sp}}_{\text{FIT}\wedge \text{MT}}=0.996$) appear to be practically relevant. For p=0.02, the triple test is optimal if ρ≥0.3, which corresponds to a benefit‐cost ratio for cancer treatment of 2.33. The side effects of colonoscopy are negligible, as the probability of needing COL after a positive result for both FIT and MT is only 0.017. For a benefit‐cost ratio between 2.33 and 20, the combined test FIT∧MT, with its higher sensitivity (0.68 vs. 0.61), becomes the optimal choice. For ratios exceeding 20, FIT alone is optimal, with a sensitivity of 0.73.

### 4.3 Stable Coronary Artery Disease Diagnostics

The European Society of Cardiology (ESC) published guidelines on the management of stable coronary artery disease (CAD) in 2013 [22]. These test guidelines differentiate according to a patient's pre‐test probability p of suffering stable CAD. The ESC task force recommended not to perform tests if p is below 15%, and non‐invasive tests in patients with p between 15% and 85%. If p exceeds 85%, the diagnosis of stable CAD should be made clinically.^9^ This recommendation is based on the observation that non‐invasive cardiac tests on average have a sensitivity and a specificity equal to about 85%. The task force argues that because 15% of test results will be incorrect, not using a test at all will lead to fewer incorrect diagnoses for patients with p<15% or p>85%. Apparently, this recommendation does not consider the cost and potential harm of testing. Furthermore, it implicitly assumes that b=l (i.e., the net utility of treating a patient with stable CAD is equal to the utility loss of treating a patient without stable CAD).^10^

In a recent publication, Min et al. (2017) analyzed single and combined test strategies for stable CAD, taking into account the cost and harm of the test and the benefit and cost of treatment. The different single tests include exercise treadmill testing (ETT), stress echocardiography (SE), myocardial perfusion scintigraphy (MPS), coronary computed tomographic angiography (CCTA), and invasive coronary angiography (ICA) [40]. The latter, however, is rather costly and comes with a 1% mortality rate. Table 3 shows the parameter values based on data from Min et al. (2017) [40]. MPS is dominated by SE and CCTA in terms of sensitivity, specificity, and cost. To calibrate the model, we set b/l=1.9 such that the test threshold for ETT is equal to 15%. At the same time, the test‐treatment threshold for CCTA becomes 87%, which is close to the ESC 2013 guidelines at which non‐invasive testing is no longer indicated. The average cost of treatment, estimated by Min et al. (2017), is l= USD 55 000 for patients with a 20% pre‐test probability of stable CAD. The benefit of treatment then is b=USD105000.

**TABLE 3:** Characteristics of ETT, SE, MPS, and CCTA for stable CAD diagnosis.

	ETT	SE	MPS	CCTA
Se	0.68	0.867	0.806	0.937
Sp	0.77	0.807	0.747	0.847
$c^{\text{Dx}}$	USD 100	USD 340	USD 819	USD 394
$c^{\text{Dx}}/b$	$9.57\times 10^{-4}$	$3.25\times 10^{-3}$	$7.84\times 10^{-3}$	$9.57\times 10^{-4}$

**FIGURE 5:**
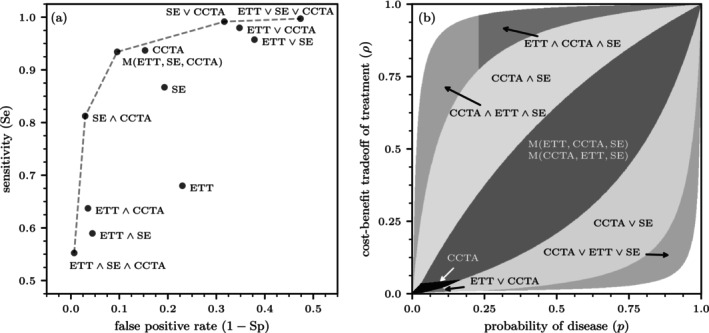
Stable coronary artery disease (CAD) diagnostics. (a) The ROC curve for CAD testing. (b) Regions within the (p,ρ) unit square where different testing protocols are optimal. In the white regions, the INB for all available test sequences is negative, indicating that no testing should be performed. The sequence of tests does not influence the sensitivity and specificity values shown in panel (a). However, it is crucial in determining the optimal testing protocols illustrated in panel (b). Because the sequence of the first two tests in the majority protocol does not affect the outcome, the protocols M(ETT,CCTA,SE) and M(CCTA,ETT,SE) are equivalent in terms of their incremental net benefits.

According to Figure 5a, five different test strategies constitute the ROC curve for stable CAD testing. The single tests SE and ETT are far off the efficient frontier. CCTA is weakly dominated. The highest sensitivity is achieved with the disjunctive triple test ETT∨SE∨CCTA, the highest specificity with the conjunctive triple test ETT∧SE∧CCTA. The double tests that exclude the inefficient ETT, that is, SE∨CCTA and SE∧CCTA, are also part of the ROC curve, as is the triple test with the majority rule.

Figure 5b shows the optimal test protocols, depending on a patient's probability of stable CAD, p, and their individual treatment threshold ρ. The single tests ETT and SE are never optimal. CCTA is the best option if both p and ρ are low, specifically when p<18% and 1%<ρ<3%. If ρ<1%, the disjunctive double test ETT∨CCTA can be the preferred choice. CCTA would only be used if ETT is negative. Despite its insufficient test accuracy, ETT is used first because it is much less costly than CCTA.

The task force also published testing ranges for individual test options. If the patient is suitable and the technology and local expertise are available, the ESC guidelines recommend the use of CCTA in patients with low to intermediate p of 15%–50%. Alternatively, for patients with p between 15% and 85%, stress imaging testing (SE, MPS, SPECT, PET) is advised. If we follow the task force's implicit ρ=0.5, CCTA is optimal for p up to 50%, although not as a single test, but in conjunctive combination with SE. CCTA again is optimal for high p, now in disjunctive combination with SE. Changes in ρ, including down to 35%, which follows from b/l=1.9, will not change the optimal test strategies as a function of p much. Given the relatively wide range of p for patients with stable CAD, the majority functions M(ETT,CCTA,SE) and M(CCTA,ETT,SE) may be optimal, depending on the values of p and ρ. Because the sequence of the first two tests in the majority protocol does not affect the outcome, the protocols M(ETT,CCTA,SE) and M(CCTA,ETT,SE) are equivalent in terms of their incremental net benefits.

## 5 Discussion

We studied the optimal aggregation of results from multiple diagnostic tests, using the incremental net benefit (INB) to quantify the trade‐offs between the informational value of the tests, test costs, and the associated benefits and harms of treatment. An online tool that visualizes the INB for various combined tests and parameters is available at https://optimal‐testing.streamlit.app/.

Consistent with previous work on the aggregation of the results of multiple tests [7, 23], our findings confirm that the receiver operating characteristic (ROC) curve is useful for evaluating tests based on their informational value. However, an efficient test (i.e., one located on the ROC frontier) may not be optimal for a specific medical application, as optimality requires maximizing the INB, which depends on both the test's informational value and health‐economic factors. Likewise, tests that are inefficient from an informational perspective may still be optimal due to their low costs and minimal side effects.

Using three application examples focused on prostate cancer, colorectal cancer, and stable coronary artery disease diagnostics, we identify decision boundaries that determine when different combinations of tests are optimal, based on a patient's pre‐test probability of disease and their cost‐benefit trade‐off from treatment. For prostate cancer diagnostics, the most commonly used tests include the free‐to‐total prostate specific antigen (PSA) test and its combination with the human kallikrein 2 (hK2) marker, where hK2 is performed first, and the PSA test is performed if the hK2 result is positive. However, the benefit‐cost ratio of a biopsy in case of positive test outcomes needs to be 10 to justify the use of the combined double test and even 24 for the single hK2 test. The implied small range for testing for prostate cancer is due both to the low accuracy of these tests and the low probability of developing the disease. For colorectal cancer, the single fecal immunochemical test and conjunctively combined triple tests are of particular practical relevance due to the disease's low prevalence. In contrast, for stable coronary artery disease, a broader range of tests, including the single coronary computed tomographic angiography test, conjunctively and disjunctively combined triple tests, and majority protocols, is practically relevant due to the condition's wider prevalence range.

Three limitations are worth noting. First, we assume that the outcomes of different tests are conditionally independent, given the disease status. This assumption is widely used in the medical decision‐making literature as it simplifies the mathematical analysis of aggregated test results. In addition, manufacturers typically report performance measures for individual tests without addressing potential dependencies between them. However, in practice, test results may show correlations. Second, while we used established estimates for parameters such as test costs and the benefits and harms of treatment, these parameters may vary in practice due to heterogeneous population effects and other context‐specific factors. Similarly, test characteristics (sensitivity and specificity) reported in the literature may be subject to spectrum bias [41]. For example, the sensitivity and specificity estimates for FT and hK2 used in this work were derived from a cohort of men with elevated PSA levels [20], who are at higher risk for prostate cancer. Their diagnostic accuracy may be lower in lower‐risk populations, which would make these tests less attractive for broader use. Third, when a test is based on a continuous marker (e.g., a measured PSA value), the outcome of an individual test must be determined by selecting an appropriate cutoff value. The optimal cutoff can be derived using the INB(p,ρ) framework [12]. Interestingly, when all tests rely on markers, the standard positivity criteria such as AND, OR, and Majority are no longer applicable. However, the INB approach remains useful in such cases. For an application on the triple test in breast cancer screening, see Felder et al. (2003) [42].

The limitations described present valuable opportunities for future research. Quantifying the effects of correlations between test results and obtaining more accurate estimates of the parameters involved in the INB calculation can contribute to further improving medical decision‐making processes that rely on aggregating results from multiple tests. Another interesting direction for future work is to study the applicability of our proposed methods in the monitoring and management of infectious diseases [43, 44].

## Conflicts of Interest

The authors declare no conflicts of interest.

## Supporting information


**Data S1**: Supporting Information.

## Data Availability

All data used are presented in the paper. See data file.pdf that gives information on how to access the APP (https://optimal‐testing.streamlit.app/) and the code https://github.com/lubo93/optimal_testing/tree/main. It includes all INB calculations.

## ENDNOTES

^1^
For comparing two therapies, the INB is used as an alternative to the incremental cost‐effectiveness ratio (ICER) (see Willan and Lin (2001) [29] for an early contribution).

^2^
We express the utility gains and losses of treatment and testing in monetary terms to make them commensurate with cost. QALY is a standard measure used to quantify and compare different dimensions of health outcomes [30], and λ is the conversion factor between QALY and monetary cost.

^3^
More precisely, ρ/(1−ρ)=l/b quantifies the utility loss of treating a healthy patient relative to the benefit of treatment if the patient is ill.

^4^
Pauker and Kassirer (1975) [24] formally derived the treatment threshold in the p domain. However, the underlying idea had already been expressed by Bleuler (1919, p. 89) [31], who noted that “the probabilities of harm and utilities must be weighed against each other in relation to their respective sizes.”

^5^
See p. 4 of Vickers and Elkin (2016) [27]. Since $p^{\text{Rx}}=\rho$ [see Equation (4)], and in line with the decision‐curve analysis, Equation (6) can be written as $\text{VI}\left(p,p^{\text{Rx}},\text{Se},\text{Sp}\right)$. Our formulation with ρ has the advantage that the cost‐benefit trade‐off involved in the treatment decision is explicit in the value of information.

^6^
For a more detailed comparison of these approaches, see Felder and Mayrhofer (2022), Chapter 5 [12]. See also Baker and Kramer (2006) [33] for an additional discussion of conceptual development.

^7^

${\text{LR}}^{+}=\text{Se}/(1-\text{Sp})$, ${\text{LR}}^{-}=(1-\text{Se})/\text{Sp}.$

^8^
If arbitrary AND/OR combinations are allowed, the tests (FT∧hK2)∨(FT∧TRUS) with Se=0.88 and 1−Sp=0.44, and FT∨(hK2∧TRUS) with Se=0.95 and 1−Sp=0.66, are efficient. As a result, FT∧TRUS would become inefficient.

^9^
ESC refined its guidelines in 2019 and 2024 [38, 39]. By and large, it confirmed the > 15%–85% non‐invasive testing range for the pre‐test probability, although it narrowed the targeted indication from “stable CAD” to “obstructive CAD”.

^10^
This can be verified if we set ${\underline{p}}^{\text{Dx}}=0.15$, ${\bar{p}}^{\text{Dx}}=0.85$ and solve for b/l [see Equations (9) and (10)].
