# Supplementary material for: Determining the Optimal Sequence of Multiple Tests
Source: Stat Med. 2025 Oct 17;44(23-24):e70254. doi: 10.1002/sim.70254 (PMC12533563; doi:10.1002/sim.70254)
Supplement: Supplementary file 1 — Data S1: Supporting Information. [file SIM-44-0-s001.pdf]

The code for the App (https://optimal-testing.streamlit.app/) is already freely accessible: https://github.com/lubo93/optimal_testing/tree/main.
It includes all INB calculations.
